# Characterization of stable hypoxia-preconditioned dental pulp stem cells compared with mobilized dental pulp stem cells for application for pulp regenerative therapy

**DOI:** 10.1186/s13287-021-02240-w

**Published:** 2021-05-29

**Authors:** Mohammed Zayed, Koichiro Iohara, Hideto Watanabe, Mami Ishikawa, Michiyo Tominaga, Misako Nakashima

**Affiliations:** 1 Research Institute, Department of Stem Cell Biology and Regenerative Medicine, National Center for Geriatrics and Gerontology, 7-430, Morioka, Obu, Aichi 474-8511 Japan; 2 Department of Surgery, College of Veterinary Medicine, South Valley University, Qena, 83523 Egypt; 3 Institute for Molecular Science of Medicine, Aichi Medical University, Nagakute, Aichi 480-1195 Japan; 4 Air Water Group, Aeras Bio Inc., Kobe, Hyogo 650-047 Japan

**Keywords:** Dental pulp stem cells, Hypoxia, Prime condition, Pulp regeneration, Dog teeth

## Abstract

**Background:**

Dental pulp stem cells (DPSCs) have been developed as a potential source of mesenchymal stem cells (MSCs) for regeneration of dental pulp and other tissues. However, further strategies to isolate highly functional DPSCs beyond the colony-forming methods are required. We have demonstrated the safety and efficacy of DPSCs isolated by G-CSF-induced mobilization and cultured under normoxia (mobilized DPSCs, MDPSCs) for pulp regeneration. The device for isolation of MDPSCs, however, is not cost-effective and requires a prolonged cell culture period. It is well known that MSCs cultured under hypoxic-preconditions improved MSC proliferation activity and stemness. Therefore, in this investigation, we attempted to improve the clinical utility of DPSCs by hypoxia-preconditioned DPSCs (hpDPSCs) compared with MDPSCs to improve the potential clinical utility for pulp regeneration in endodontic dentistry.

**Methods:**

Colony-forming DPSCs were isolated and preconditioned with hypoxia in a stable closed cultured system and compared with MDPSCs isolated from the individual dog teeth. We examined the proliferation rate, migration potential, anti-apoptotic activity, and gene expression of the stem cell markers and angiogenic/neurotrophic factors. Trophic effects of the conditioned medium (CM) were also evaluated. In addition, the expression of immunomodulatory molecules upon stimulation with IFN-γ was investigated. The pulp regenerative potential and transplantation safety of hpDPSCs were further assessed in pulpectomized teeth in dogs by histological and immunohistochemical analyses and by chemistry of the blood and urine tests.

**Results:**

hpDPSCs demonstrated higher proliferation rate and expression of a major regulator of oxygen homeostasis, *HIF-1α*, and a stem cell marker, *CXCR-4*. The direct migratory activity of hpDPSCs in response to G-CSF was significantly higher than MDPSCs. The CM of hpDPSCs stimulated neurite extension. However, there were no changes in angiogenic, migration, and anti-apoptotic activities compared with the CM of MDPSCs. The expression of immunomodulatory gene, *PTGE* was significantly upregulated by IFN gamma in hpDPSCs compared with MDPSCs. However, no difference in nitric oxide was observed. The regenerated pulp tissue was quantitatively and qualitatively similar in hpDPSC transplants compared with MDPSC transplants in dog teeth. There was no evidence of toxicity or adverse events of the hpDPSC transplantation.

**Conclusions:**

These results demonstrated that the efficacy of hpDPSCs for pulp regeneration was identical, although hpDPSCs improved stem cell properties compared to MDPSCs, suggesting their potential clinical utility for pulp regeneration.

**Supplementary Information:**

The online version contains supplementary material available at 10.1186/s13287-021-02240-w.

## Background

Dental pulp stem cells (DPSCs) are fibroblast-like adhesive cells characterized by colony-forming activities, self-renewal, and multi-lineage differentiation potential similar to other mesenchymal stem cells (MSCs) [1]. DPSCs have remarkable biological properties including high proliferation and migration abilities, and immunomodulatory and angiogenic/neurotrophic effects of their secretome [2–4]. DPSCs can be readily isolated from the discarded teeth with no ethical issues [5]. Further, they may be stored in a stem cell bank. It is noteworthy that several preclinical/clinical studies have demonstrated the therapeutic potential of DPSCs for regeneration of various tissue diseases including ischemic brain injury, infarcted myocardium, muscular dystrophy, and in dentistry [6, 7].

The DPSCs have been isolated and cultured typically by colony-formation method [2]. We demonstrated that the regenerated pulp tissue was less in volume and mineralized after transplantation of unfractionated DPSCs compared with fractionated DPSC subfraction of CD105^+^ cells in the pulpectomized dog teeth [8]. The isolation methods by the flow cytometer or magnetic cell selection system device using the stem cell marker CD105 were not cost-effective in safety and efficacy for manufacturing clinical grade DPSCs. Thus, we have developed a novel isolation method based on ability of DPSC subfractions to mobilized by granulocyte colony-stimulating factor (G-CSF) [9]. The mobilized DPSCs (MDPSCs) showed higher expression of stem cell markers, higher trophic effects on anti-apoptosis, migration, angiogenesis, neurite extension, and immunomodulation, and higher pulp regenerative potential [9, 10]. Our clinical study further demonstrated the clinical grade human MDPSCs are safe and efficacious for complete pulp regeneration [11]. The device for isolation of MDPSCs, however, is not cost effective and the cell culture period is prolonged. Thus, the cost-effective method and safety of the isolation and processing of good manufacturing practice (GMP) grade DPSC subsets with high-regeneration potential remains a challenge, and we have addressed this in the present investigation.

Significant attempts have been made to modify the microenvironment of directed tissues through management of MSC behavior and outcome in vitro by seeding density, passage number, coating surfaces, and three-dimensional scaffolds [12]. Moreover, preconditioning with specific biological factors or cytokines, genetic modification, and hypoxic treatment have been suggested to improve MSC properties [13]. Above all, oxygen (O_2_) concentration is one of the most important critical factors to play an ultimate role in cell growth and metabolism. Currently, in vitro cultures of MSCs are typically done in a 95% air supplemented with 5% of carbon dioxide (CO_2_). The endogenous physiological oxygen concentration is critical for the optimal outcome of cell growth and differentiation [14]. There are many studies reported a negative impact of ambient O_2_ concentration on cultured MSCs, including decreased proliferation rate, DNA damage, and early senescence [15, 16]. On the other hand, hypoxia was reported to have a profound effect on MSCs to increase proliferation rate [15], plasticity [17], engraftment [18], reduction of reactive oxygen species [19], and expression of chemokine receptors and migration [20]. A range of 3 to 6% O_2_ (20 to 40 mmHg) has been identified in a physiological state of adult organs and tissues [21]. The definite oxygen concentration in situ, however, varies mostly on the vascularization and metabolic activity of the tissue [22]. The dental pulp has a relatively high blood flow, well irrigated, giving a range of 2 to 6% of partial pressure of oxygen level [23]. We recently demonstrated that low oxygen supplementation (5%) is ultimate to enhance proliferation rate, stem cell properties, and trophic effects of secretome in cultured DPSCs [24]. We further developed a closed culture system in which only one octahedron container was used for expansion from the primary to the third passage of culture with the stable oxygen concentration and pH. Our preliminary results demonstrated that the colony-forming human DPSCs proliferated better both in 5% O_2_ and 3% O_2_ compared to those in 20% O_2_ condition and the stem cell properties of DPSCs cultured between 5% O_2_ and 3% O_2_ conditions were similar.

The aim of this study was to examine whether DPSCs isolated and preconditioned with stable 5% O_2_, named hpDPSCs could have optimal stem cell properties and pulp regenerative potential. Thus, we attempted to develop the cost-effective and safe methods for improving GMP-grade cell processing of DPSC subsets instead of G-CSF-induced mobilization method. We evaluated proliferation rate, migration activity, gene expression of stem cells markers, immunomodulatory, and trophic factors. The various trophic effects of the CM were also examined and compared to MDPSCs. Furthermore, pulp regenerative potential compared to MDPSCs and transplantation safety of hpDPSCs were examined in pulpectomized teeth in dogs. These analyses have led us to propose that the hpDPSCs could be used as a potential clinical replacement of the MDPSCs for optimal pulp regenerative cell therapy.

## Methods

### Culture of hpDPSCs

All animal procedures were approved by the Animal Care and Use Committee of the National Center for Geriatrics and Gerontology, Research Institute (permission #30-19, #31-17) and the Aichi Medical University (permission #2019-92, #2020-92) and Shin Nippon Biomedical Laboratories Ltd (permission #IACUC860-017). All procedures and methods were performed in accordance with relevant guidelines and regulations. Upper third incisors, a total of 12 teeth from 6 young female beagle dogs (Kitayama Lab, Ina, Japan) at 1 year old were used for isolation of MDPSCs and hpDPSCs. For hpDPSCs, isolated DPSCs were cultured in a stable hypoxic condition in a closed container with a regular octahedron having 21 cm^2^ of each surface (Animal Stem Cell, Tokyo, Japan), in which humidified gas mixtures of the composition of 5% O_2_–5% CO_2_–90% N_2_ were flushed. The pH of the hypoxic cultures was adjusted by adding HEPES buffer (Gibco, Dublin, Ireland) at a final concentration of 25 mM to Dulbecco’s modified Eagle’s medium (DMEM) (Sigma Aldrich, MO, USA) supplemented with 10% fetal bovine serum (FBS, GE Healthcare, Little Chalfont, England). Each sensor chip (SP-LG1-SA-S, and SP-PSt3-SA, PreSens, Regensburg, Germany) was patched on the bottom of the container inside, respectively, and the pH and O_2_ concentration in the DMEM and the air were measured by non-contact pH meter (pH-1SMA LG1; PreSens) and non-contact oxygen analyzer (OXY-1SMA trace, PreSens).

The primary colony-derived DPSCs were expanded in the same one surface of the octahedron container at the 2nd passage of culture and further cultured in the all surface of the container by rotating 45° every 1 min by a rotary equipment (Biomedica Solution, Ibaraki, Japan) at the 3rd passage. These hpDPSCs were detached and cryopreserved at 1 × 10^6^ cells /mL in the stem cell banker (ZENOAQ Co., LTD., Fukushima, Japan) for further experiments.

MDPSCs based on their migratory response to G-CSF (Neutrogin, Chugai Pharmaceutical, Tokyo, Japan) were isolated at the 2nd passage from colony-derived primary DPSCs and cultured according to our previous study [9]. In brief, colony-derived primary DPSCs were seeded into the upper chambers (permeable support 8.0 μm polycarbonate membrane 6.5 mm Insert, Corning, Lowell, MA) which inserted in 24 well plate contained with DMEM supplemented with 10% FBS and 100 ng/ml G-CSF. The membrane was modified chemically (Toray Industries, Co., Ltd., Tokyo) to prevent cell attachment. After 48 h, the chamber was removed, and medium was changed into DMEM with 10% FBS. Once cells reached 60–70% confluence, they were detached and subcultured.

### Doubling time

The population doubling time was calculated by counting the cell number from the 2nd expansion to the 3rd expansion. The cells were stained with trypan blue and the viable cells were counted with a hemocytometer.

### Real-time reverse transcription-polymerase chain reaction analysis

For real-time reverse transcription-polymerase chain reaction (RT-PCR) analysis, total RNA was extracted using TRIzol (Thermo Fisher Scientific, Waltham, MA, USA) from hpDPSCs and MDPSCs at the 5th to 6th passage of culture. First-strand cDNA syntheses were performed from 1 μg of total RNA by reverse transcription using ReverTra Ace-α (Toyobo, Tokyo, Japan). Real-time PCR amplifications were performed using canine HIF-1α (forward) 5′-ACTGATGACCAACAACTTGAGG-3′ and (reverse) 5′-TTTGGAGTTTCAGAAGCAGGTA-3′. Canine stem cell markers, angiogenic/neurotrophic factors, and immunomodulatory factors were used as our previous studies [8, 25]. All primers were labeled with Power SYBR Green PCR Master Mix (Applied Biosystems, Foster City, CA, USA) in 7500 RT-PCR system (Applied Biosystems). The relative mRNA expression was examined in hpDPSCs to MDPSCs after normalizing with β-actin.

### Migration activity of hpDPSCs and MDPSCs

To determine the migratory activity in response to G-CSF, 1 × 10^5^ of hpDPSCs or MDPSCs were seeded in 100 μl of DMEM on top of an insert membrane with 8-μm pore size in 24-well plates (Corning-Transwell- polycarbonate membrane cell culture inserts, Sigma-Aldrich, Missouri, USA). The lower compartment medium contained 2% FBS and was supplemented with or without G-CSF (100 ng/ml). After 24 h, cells were removed from the top of the membrane with cotton swabs. The migrating cells on the lower surface of the membrane were fixed with 95% methanol and stained with 0.5% Giemsa stain for 15 min. After washing, the stained cells were counted in 4 fields per well under an inverted bright-field microscope (Leica, 6000B-4, Leica Microsystems GmbH, Wetzlar, Germany) at × 100 magnification.

### The effect of the CM on angiogenesis and neurite extension

For collecting the conditioned media (CM), the hpDPSCs and MDPSCs were cultured in the complete culture medium. The medium was changed into DMEM without serum at 70% confluence, and the CM collected 48 h later and concentrated by Amicon Ultra-15 Centrifugal Filter (Millipore, Billerica, MA, USA). To compare the stimulative effect of the CM on endothelial cell differentiation, human umbilical vein endothelial cells (HUVEC, clone 7F3415, Lonza) were seeded on Matrigel (BD Biosciences, San Jose, CA, USA) in DMEM containing 2% FBS, 5 μg/ml heparin (Lonza, Basel, Switzerland), 5 μg/ml ascorbic acid (Lonza), and 5 μg/ml hydrocortisone (Lonza) supplemented with the CM (5 μg/ml proteins). The mean length of networks of cords and tube-like structures was measured 5 h after cultivation under the inverted microscope (Leica) using ImageJ software (version 1.52, imagej.nih.gov). The same experiment was performed with 100 ng/ml G-CSF (Peprotech, London, UK) as a positive control.

For examining the effect on neurite outgrowth, human neuroblastoma cell line (TGW, clone JCRB 0618, Health Science Research Resources Bank, Japan) was cultured without serum overnight and then stimulated with the CM (5 μg/ml proteins) for 24 h. The mean neurite length was measured under the inverted microscope using ImageJ software (version 1.52, imagej.nih.gov). The same experiment was performed with a 50 ng/ml neurotrophin-3 (Peprotech, London, UK) as a positive control.

### The combinatorial effect of the CM with G-CSF on migration

The migratory effects of the CM of hpDPSCs or MDPSCs together with G-CSF were compared with those of the CM only. Periodontal ligament cells (PDLCs) from young dog (10 months old) were isolated according to the previous study [26]. PDLCs were cultured in DMEM supplemented with 10% FBS and cryopreserved at the 4th to 7th passage of culture. For the migratory activity, 1 × 10^5^ of PDLCs were seeded in 100 μl of DMEM on top of the insert membrane. The lower compartment medium containing 2% FBS were supplemented with 5 μg/ml CM with or without 100 ng/ml of G-CSF. G-CSF only and 2% FBS only were used as a positive control and as a negative control respectively. After 24 h, the migrating cells were stained as previously described.

### Anti-apoptotic activity of the CM

To examine the anti-apoptotic effect of G-CSF, hpDPSCs or MDPSCs were incubated with 500 nM staurosporine (Sigma) in DMEM supplemented with 100 μg/ml of G-CSF. After 3 h, cells were harvested and the activity of caspase-3 was measured using APOPCYTOTM Caspase-3 Colorimetric Assay Kit (Medical and Biological Laboratories, Nagoya, Japan) according to the manufacturer’s protocol.

For examining the combinatorial effect of the CM with G-CSF on anti-apoptotic activity, canine PDLCs were cultured in DMEM with staurosporine and 50 μg/ml of CM with or without 100 ng/ml of G-CSF and the activity was measured as previously described.

### Immunomodulation activity upon stimulating with interferon gamma (IFN-γ)

hpDPSCs and MDPSCs were stimulated with IFN-γ (PROSPEC, East Brunswick NJ, USA) at a concentration of 20 ng/ml in DMEM without serum for 24 h according to the previous studies [27, 28] with slight modification. Non-stimulated cells were used as a control. RNA was extracted using Trizol, and the CM was collected and concentrated. The mRNA expression of immunosuppressive markers *IDO*, *TGF-β1*, *PTGE*, and *IL-6* as our previous study [29] was examined by RT-PCR. The concentration of nitric oxide (NO) was examined by measuring its stable end product, nitrite, in the CM using a Griess reagent (Promega Corporation, Madison, WI, USA) according to manufacturer’s protocol. Absorbance at 540 nm was measured by microplate reader (SpectraMax Gemini XPS/EM, Molecular Devices, San Jose, CA, USA), and nitrite concentrations were calculated using a standard nitrite curve.

### Comparison of trophic factor mRNA expression between the rotating and stationary conditions

After validation the pH and O_2_ concentration, we examined the effect of the rotating culture. Freshly isolated pulp cells from an upper fourth incisor from 1-year-old dog were plated into three containers, and the cultured hpDPSCs were further divided into the two containers at the 3rd passage of culture, respectively; 1/9 of the total cells was plated in the stationary condition at one surface of the octahedron closed container with 5% O_2_ and the remaining 8/9 was plated in the rotating condition at eight surfaces of the container with 5% O_2_. The trophic factor mRNA expression was compared by RT-PCR between the stationary and rotating conditions.

### Transplantation of hpDPSCs and MDPSCs into pulpectomized teeth in dogs

The upper first and second incisors, a total of 12 teeth from 3 young female beagle dogs (Kitayama Lab, Ina, Japan) at 1 year old were used. Transplantation of hpDPSCs or MDPSCs at 5 × 10^5^ cells together with G-CSF (Neutrogin) in 20 μl of atelocollagen scaffold (1% atelocollagen implant; Koken, Tokyo, Japan) was performed for pulp regeneration in the pulpectomized teeth as described previously with slight modification [10]. The teeth were extracted at 4 weeks after cell transplantation. Histological examination of the regenerated tissue was performed in the paraffin sections (5 μm in thickness) of the teeth. The regenerated tissue was outlined in on-screen image of the histological preparations of each 4 sections (*n =* 6) by a binocular microscope (Leica, M 205 FA Leica Microsystems, Wetzlar, Germany), and its relative amount to the root canals was determined by using Leica Application Suite software (Leica, version 3.4.1). For neovascularization and innervation analyses, Fluorescein Griffonia (Bandeiraea) Simplicifolia Lectin 1/fluorescein-galanthus nivalis (snowdrop) or anti-PGP9.5 (Ultra Clone) (1: 10,000) were used, respectively. The ratios of newly formed capillary area and neurite extension area to the regenerated pulp area were measured respectively by Dynamic cell count BZ-HIC (KEYENCE, Osaka, Japan).

For evaluation of safety of the hpDPSC transplantation, each upper left second incisor was extracted from 3 dogs at 9 to 12 months old in the Shin Nippon Biomedical Laboratories Ltd. and transported to the National Center for Geriatrics and Gerontology to isolate and culture hpDPSCs. The cryopreserved hpDPSCs were transported by air to the animal facility in the Shin Nippon Biomedical Laboratories Ltd. and autologously transplanted at 5 × 10^5^ cells into pulpectomized upper right second incisors (*n* = 3) as described previously. The dogs were observed in clinical signs, daily food consumption, and weekly weight change for toxicology assessment. Urinalysis was performed by Clinitek AtlasXL (Sparton Medical Systems, Strongsville, OH, USA) at 2 and 4 weeks. Blood tests and blood chemistry examinations were performed by ADIVIA 120 (Siemens Healthcare Diagnostics Manufacturing Ltd., Erlangen, Germany) and by JCA-BM6070 (Japan Electron Optical Laboratory, Tokyo, Japan), respectively, at 1, 2, and 4 weeks. One dog without cell transplantation was used as a control. The transplanted teeth were extracted, and all organs were weighed and macroscopically examined at 4 weeks followed by euthanization. They were further examined histopathologically in the paraffin sections stained with hematoxylin and eosin (HE).

### Statistical analysis

All the results were expressed as the means ± standard deviation (SD). Student’s *t* test was used for a two-group comparison. For the migration, angiogenesis, neurite extension, anti-apoptotic, and immunomodulation assays, a one-way analysis of variance (ANOVA) was used followed by a Tukey’s comparisons post hoc test using SPSS 25.0 (IBM, Armonk, NY). A *p* value less than 0.05 was considered statistically significant.

## Results

### Maintenance of pH and O_2_ concentration

The pH and O_2_ concentrations of the hypoxic cell cultures were monitored for 29 days, demonstrating that pH value was maintained between 6.85 and 7.01 and O_2_ concentration value was maintained at 5.4–6.5 and 5.1–5.9 in the DMEM liquid and in the air of the container, respectively (Supplemental Fig. 1).

### Decreased doubling time of hpDPSCs

**Fig. 1:**
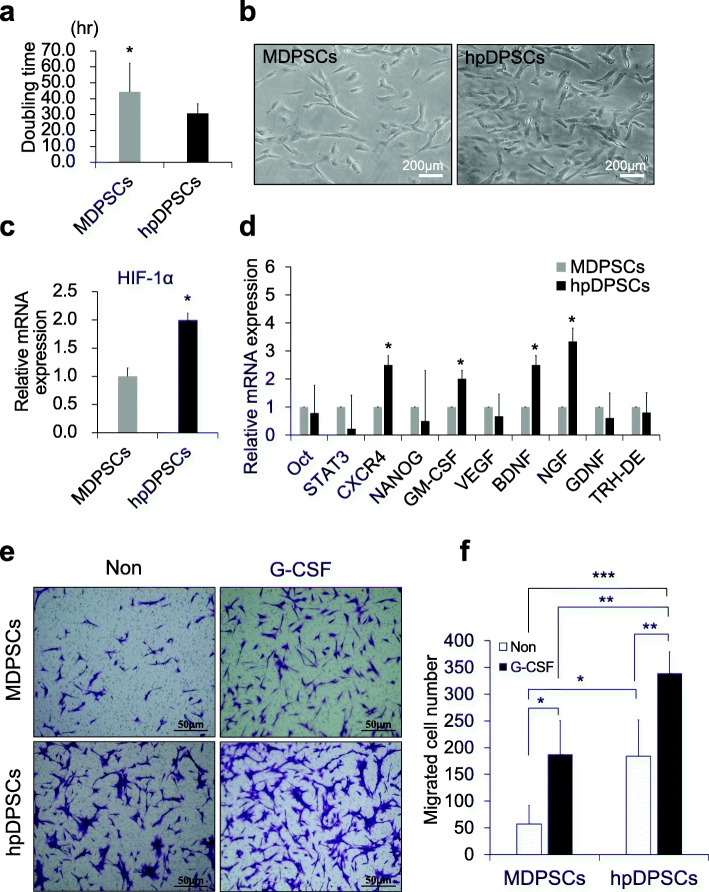
Stem cell properties of hpDPSCs compared with MDPSCs. **a** Proliferation activity, doubling time. *p < 0.05. **b** A representative images of the morphology. **c**, **d** Gene expression of HIF-1α, stem cell markers, trophic factors, and immunomodulatory markers. *p < 0.05. **e** Migration activity in response to G-CSF. **f** Statistical analysis of migration activity after 24 h. *p < 0.05, **p < 0.01, and ***p < 0.001. All data are expressed as the means ± standard deviation (*n* = 3)

The doubling time of hpDPSCs was significantly lower compared to MDPSCs (*p* < 0.05) (Fig. 1a). There was little difference in the cell morphology between hpDPSCs and MDPSCs, showing stellate or spindle-shaped morphology at the 3rd passage of culture (Fig. 1b). The total culture period of hpDPSCs was shorter (12 ± 0.5 days) compared with that of MDPSCs (20 ± 2 days).

### Expression of stem cell markers, trophic factors, and immunomodulatory genes

The mRNA expression levels of a major regulator of oxygen homeostasis, *HIF-1α*, were two times higher in hpDPSCs compared to MDPSCs (*p* < 0.05) (Fig. 1c), indicating hypoxic effect. A stem cell marker, *CXCR4* was also significantly higher in hpDPSCs (Fig. 1d), suggesting more enrichment of the stem cells or establishing of the stem cell properties. However, other markers *Oct4*, *STAT3*, and *NANOG* were similarly expressed. Expression of angiogenic factor, *GM-CSF* was significantly higher in hpDPSCs compared to MDPSCs (*p* < 0.05). However, *VEGF* expression was similar. Moreover, expression of neurotrophic factors, *BDNF* and *NGF* were significantly higher in hpDPSCs (*p* < 0.05), but not *GDNF* (Fig. 1d). In addition, the expression level of a pulp marker, *TRH-DE*, was similar between hpDPSCs and MDPSCs (Fig. 1d).

### Enhanced migration activity of hpDPSCs

Next, the migration activity of hpDPSCs and MDPSCs was examined. hpDPSCs were able to migrate at the higher rate without G-CSF compared to MDPSCs (*p* < 0.05) (Fig. 1e, f). It is noteworty that the migration of hpDPSCs with G-CSF was significantly higher compared with MDPSCs with G-CSF (*p* < 0.01) (Fig. 1f). There were also significant differences between 2% FBS only as a control and 2% FBS together with G-CSF both in hpDPSCs and MDPSCs (*p* < 0.01, and *p* < 0.05, respectively) (Fig. 1e, f). These results suggested that hpDPSCs might have the higher G-CSF receptor expression than MDPSCs.

### Higher effect of hpDPSC CM on neurite extension, comparable effect on angiogenesis and migration

**Fig. 2:**
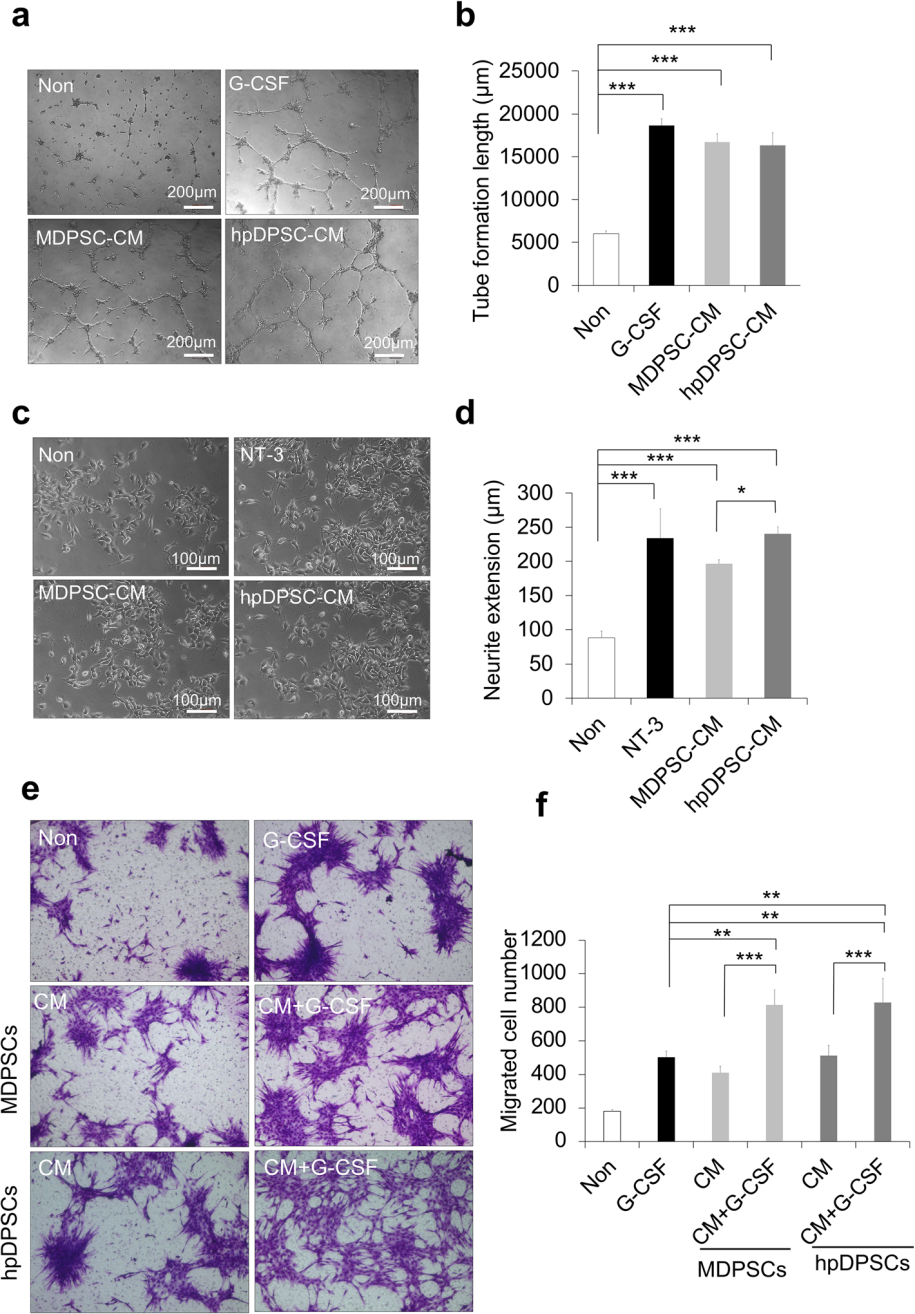
Trophic effects of the conditioned medium (CM) of hpDPSCs compared with MDPSCs. **a** Enhanced effect of the CM on angiogenic activity of HUVEC, showing network formation after 5 h. **b** Statistical analysis of the total tube length. *p < 0.05 and ***p < 0.001. **c** Stimulatory effect of the CM on neurite outgrowth of TGW cell line. **d** Statistical analysis of neurite length in the different conditions. **e** Migration activity of periodontal ligament cells toward the CM with or without G-CSF (100 ng/ml). **f** Statistical analysis of migration activity after 24 h. **p < 0.01 and ***p < 0.001. All data are expressed as the means ± standard deviation (n = 3)

The CM of hpDPSCs and MDPSCs was further used to examine enhanced angiogenic and neurite extension activities. There was no difference in the angiogenic activity between the hpDPSC CM and the MDPSC CM, although the angiogenic activities of those CM were significantly higher compared with control (Fig. 2a, b). On the other hand, the hpDPSC CM demonstrated a significantly higher stimulatory effect on neurite outgrowth in human neuroblastoma TGW cells than the MDPSC CM (*p* < 0.05) (Fig. 2c, d).

The effect of the hpDPSC CM and the MDPSC CM both together with G-CSF on migration activity was further examined in canine PDLCs. Both the CM with G-CSF showed the higher migration activities compared to the CM only (*p* < 0.001) and G-CSF only (*p* < 0.01) (Fig. 2e, f). However, no significant difference between the hpDPSC CM and the MDPSC CM, and between the hpDPSC CM with G-CSF and the MDPSC CM with G-CSF was observed (Fig. 2e, f). These results demonstrated the similar combinatorial effect of the hpDPSC CM to the MDPSC CM together with G-CSF.

### Comparable effect of hpDPSC CM on anti-apoptosis

**Fig. 3:**
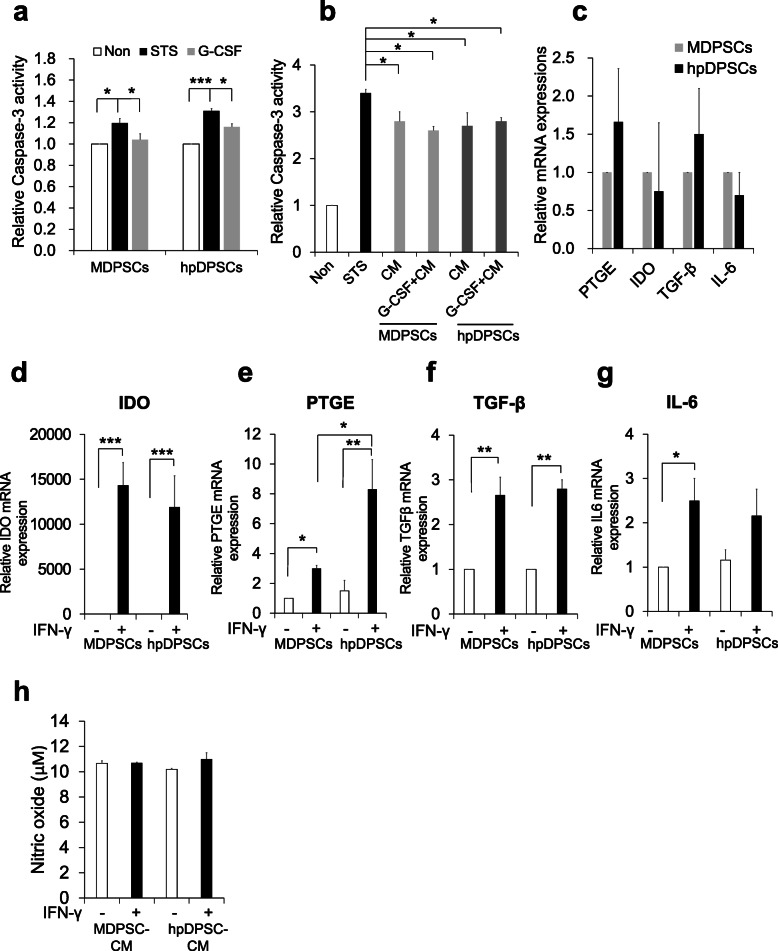
Anti-apoptotic and immunomodulatory activities of hpDPSCs and MDPSCs. **a** Anti-apoptotic activity showing Caspase-3 activity of hpDPSCs and MDPSCs in response to G-CSF. **b** Anti-apoptotic effect of hpDPSC and MDPSC CM in the staurosporine-treated canine PDLCs. **c** Immunomodulation activity of hpDPSCs and MDPSCs under homeostasis condition. **d**–**h** Immunomodulatory activity of hpDPSCs and MDPSCs when stimulated with IFN-γ. **d** *IDO*, **e** *PTGE*, **f** *TGF-β*, and **g** *IL-6* mRNA expression. **h** Nitric oxide in the hpDPSC CM and the MDPSC CM. *p < 0.05, **p < 0.01, and ***p < 0.001. All data are expressed as the means ± standard deviation (*n* = 3)

We examined the anti-apoptotic effect of G-CSF in the hpDPSCs compared with the MDPSCs by quantifying caspase-3 activity. The increased caspase-3 activities after treatment with staurosporine were similarly reduced by supplement with G-CSF in the hpDPSCs to the MDPSCs (Fig. 3a). The in vitro trophic effects of hpDPSC CM and MDPSC CM with or without G-CSF on anti-apoptosis were further assessed. Both the CM with or without G-CSF significantly inhibited apoptosis in the staurosporine-treated canine PDLCs (*p* < 0.05) (Fig. 3b), without showing any significant difference between the hpDPSC CM and the MDPSC CM with and without G-CSF.

### Comparable effect of hpDPSC CM on immunomodulatory activity

Under the hemostatic condition, the expression of immunosuppressive factors, *IDO*, *PTGE*, and *TGF-β* was no significant difference between in hpDPSCs and MDPSCs (Fig. 3c). To address whether priming with IFN-γ could regulate the immunomodulation of hpDPSCs and MDPSCs, we further stimulated the cells with IFN-γ for 24 h. The immunosuppression genes *IDO*, *PTGE*, and *TGF-β* were significantly upregulated both in the stimulated hpDPSCs and MDPSCs compared with those in the unstimulated (Fig. 3d–g). However, there was no significant difference between hpDPSCs and MDPSCs except for *PTGE*. The expression of *PTGE* was 2.7 times higher in hpDPSCs compared with MDPSCs (*p* < 0.05) (Fig. 3e). Production of NO in the hpDPSC CM and the MDPSC CM did not show any changes when stimulated with IFN-γ (Fig. 3h). These results demonstrate that hpDPSCs and MDPSCs have a similar immunosuppressive function when stimulated with proinflammatory cytokines.

### Little effect of rotating condition on trophic factor expression

There was little effect of rotating condition on the trophic factor expression in hpDPSCs compared with the stationary condition (Table 1). These results suggested that the present findings of the improved stem cell properties of hpDPSCs were due to hypoxic condition, not due to rotating condition.

**Table 1:** The effect of the rotating condition on the trophic factor expression compared with the stationary condition in hpDPSCs by RT-PCR

	Rotating/stationary
*GM-CSF*	1.3 ± 0.4
*CXCR4*	1.0 ± 0.4
*NGF*	1.9 ± 0.9
*BDNF*	0.9 ± 0.2
*IDO*	0.5 ± 0.2
*PTGE*	0.9 ± 0.1
*TGF-β1*	1.1 ± 0.2
*IL-6*	0.9 ± 0.1

All data are expressed as the means ± SD (*n* = 3). The experiment was repeated three times, and one representative experiment is presented

### Similar pulp regenerative potential

**Fig. 4:**
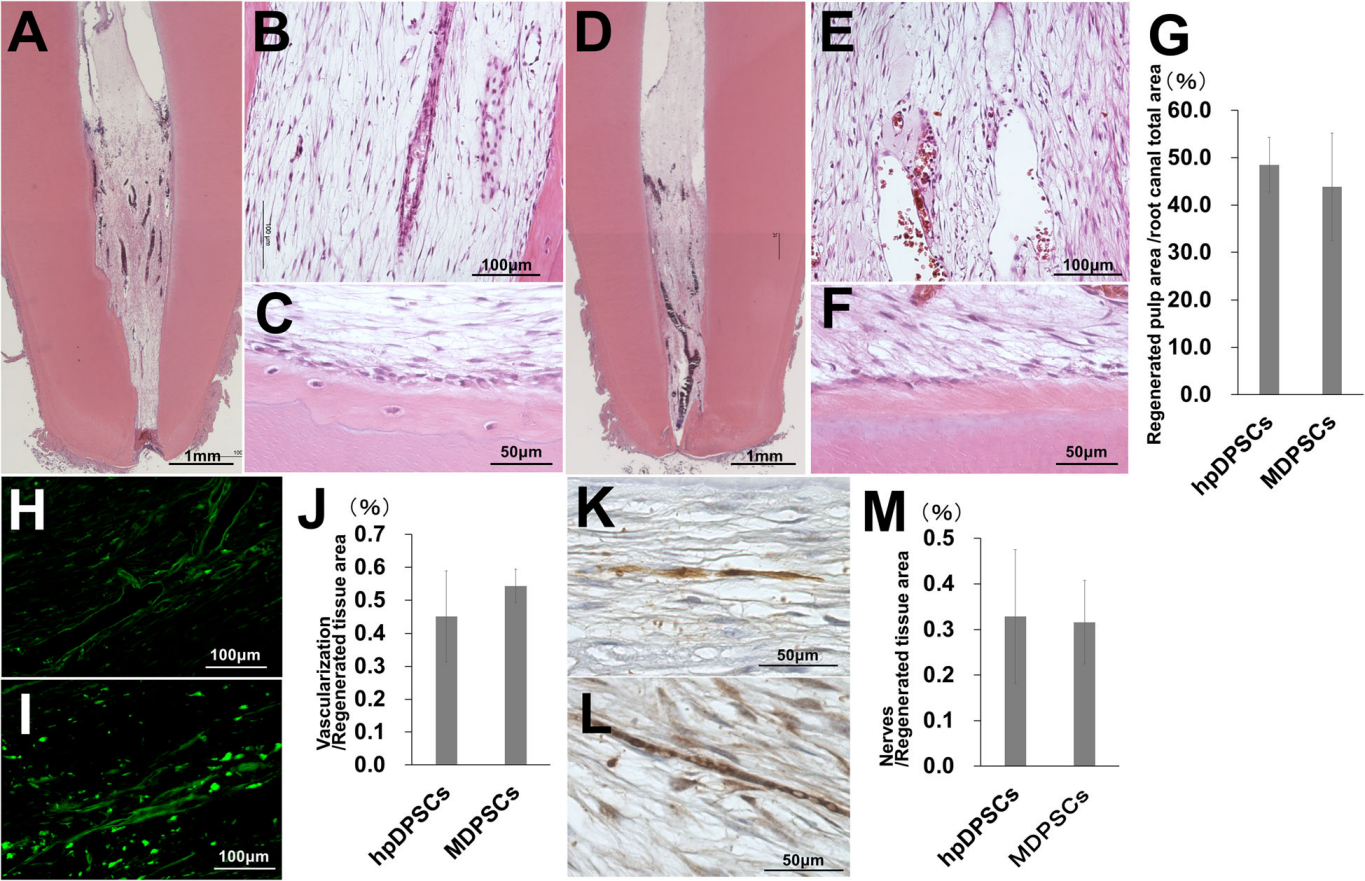
Pulp regenerative potential of hpDPSCs compared with MDPSCs at 4 weeks after cell transplantation in the dog pulpectomized teeth. **a**–**e** Pulp tissue regeneration. **c**, **f** Osteodentin/tubular dentin-like mineralized tissue formation along the dentinal wall. **h**, **i** BS1-lectin staining for vasculogenesis. **k**, **l** PGP9.5 staining for neurite extension. Statistical analysis of **g** the regenerated pulp, **j** vascularization, and **m** re-innervation. **a**–**c**, **h**, **k** The hpDPSC transplants. **d**–**f**, **i**, **l** The MDPSC transplants. All data are expressed as the means ± standard deviation (*n* = 3)

Next, the difference in a pulp regenerative potential between the hpDPSCs and the MDPSCs was examined in the dog pulpectomized teeth. The morphologically similar pulp tissue (Fig. 4a, b), well-vascularized (Fig. 4h), and well-innervated (Fig. 4k) loose connective tissue was regenerated at 4 weeks after hpDPSC transplantation with G-CSF as shown after MDPSC transplantation (Fig. 4d, e, i, l). There was little infiltration of inflammatory cells and no internal absorption (Fig. 4a, d). The osteoblastic cells confined in the mineralized tissue along the dentinal wall, and/or odontoblastic cells aligning to the newly formed mineralized tissue were observed both in the transplants of the hpDPSCs and the MDPSCs (Fig. 4c, f). There was no significant difference in the ratio of the regenerated pulp area to the total root canal area between the hpDPSC transplants and the MDPSC transplants (Fig. 4g). Furthermore, no significant difference in neovascularization (Fig. 4j) and re-innervation (Fig. 4m) was demonstrated between the hpDPSC transplants and the MDPSC transplants.

### No adverse effects

No adverse effects on appearance, clinical signs, food consumption, and body weight were detected by toxicology assessment for 4 weeks after the hpDPSC transplantation. Also, no increase of white blood cell and platelet numbers (Table 2) was observed indicating no immunoreaction toward the transplanted cells. Serum and urine chemistry parameters showed values within normal ranges for 4 weeks (Table 2). No abnormalities were observed in any organ or tissues assessed by histopathological examinations at 4 weeks. These results demonstrate that hpDPSC transplantation is safe for pulp regeneration.

**Table 2:** Safety evaluation by blood tests and blood chemistry examinations at 1, 2 and 4 weeks and urinalysis at 2 and 4 weeks after the transplantation of hypoxia-preconditioned dental pulp stem cells (hpDPSCs)

**Blood tests**																								
**Individual number**	**RBC (10**^**6**^**/μL)**				**WBC (10**^**3**^**/μL)**				**Platelet count (10**^**3**^**/μL)**				**Hematocrit (%)**											
	**pre**	**7**	**14**	**28**	**pre**	**7**	**14**	**28**	**pre**	**7**	**14**	**28**	**pre**	**7**	**14**	**28**								
**control**	**6.83**	**6.66**	**6.28**	**6.73**	**10.61**	**10.41**	**10.79**	**15.77**	**213**	**200**	**183**	**233**	**45.2**	**44.2**	**42.1**	**44.0**								
**1**	**6.38**	**6.54**	**6.16**	**5.86**	**9.13**	**10.04**	**9.91**	**9.37**	**230**	**223**	**260**	**235**	**41.9**	**42.8**	**40.6**	**38.8**								
**2**	**6.62**	**6.83**	**6.88**	**6.20**	**10.40**	**9.75**	**11.26**	**10.00**	**296**	**302**	**290**	**264**	**42.7**	**44.0**	**44.2**	**40.1**								
**3**	**7.12**	**6.73**	**6.94**	**7.51**	**8.06**	**8.49**	**8.72**	**8.50**	**258**	**252**	**266**	**269**	**46.2**	**44.2**	**45.3**	**48.5**								
**average**	**6.71**	**6.70**	**6.66**	**6.52**	**9.20**	**9.43**	**9.96**	**9.29**	**261.33**	**259.00**	**272.00**	**256.00**	**43.60**	**43.67**	**43.37**	**42.47**								
**S.D.**	**0.31**	**0.12**	**0.35**	**0.71**	**0.96**	**0.67**	**1.04**	**0.61**	**27.05**	**32.63**	**12.96**	**14.99**	**1.87**	**0.62**	**2.01**	**4.30**								
**Normal range**	**5.10-7.47**				**6.49-16.61**				**229-401**				**33.95-47.67**											
**Blood Chemistry examination**																								
**Individual number**	**AST (IU/L)**				**ALT (IU/L)**				**Albumin (g/dL)**				**Globuline (g/dL)**				**Total choresterol (mg/dL)**				**Glucose (mg/dL)**			
	**pre**	**7**	**14**	**28**	**pre**	**7**	**14**	**28**	**pre**	**7**	**14**	**28**	**pre**	**7**	**14**	**28**	**pre**	**7**	**14**	**28**	**pre**	**7**	**14**	**28**
**control**	**25**	**27**	**25**	**24**	**41**	**40**	**41**	**35**	**3.2**	**3.1**	**3.1**	**2.9**	**2.2**	**2.1**	**2.0**	**2.3**	**178**	**182**	**170**	**201**	**94**	**94**	**98**	**93**
**1**	**26**	**23**	**23**	**31**	**55**	**56**	**54**	**91**	**3.0**	**2.9**	**2.9**	**2.8**	**2.1**	**2.5**	**2.2**	**2.1**	**139**	**157**	**151**	**139**	**94**	**98**	**82**	**88**
**2**	**22**	**20**	**22**	**21**	**45**	**48**	**47**	**44**	**3.3**	**3.3**	**3.4**	**3.2**	**2.2**	**2.3**	**2.1**	**2.0**	**137**	**150**	**142**	**124**	**94**	**102**	**102**	**98**
**3**	**44**	**27**	**28**	**27**	**42**	**45**	**46**	**48**	**3.4**	**3.3**	**3.3**	**3.3**	**2.1**	**2.1**	**2.0**	**2.2**	**155**	**149**	**140**	**143**	**87**	**98**	**94**	**95**
**average**	**30.7**	**23.3**	**24.3**	**26.3**	**47.3**	**49.7**	**49.0**	**61.0**	**3.2**	**3.2**	**3.2**	**3.1**	**2.1**	**2.3**	**2.1**	**2.1**	**143.7**	**152.0**	**144.3**	**135.3**	**91.7**	**99.3**	**92.7**	**93.7**
**S.D.**	**9.57**	**2.87**	**2.62**	**4.11**	**5.56**	**4.64**	**3.56**	**21.28**	**0.17**	**0.19**	**0.22**	**0.22**	**0.05**	**0.16**	**0.08**	**0.08**	**8.06**	**3.56**	**4.78**	**8.18**	**3.30**	**1.89**	**8.22**	**4.19**
**Normal range**	**19-39**				**20.8-58.8**				**2.67-3.43**				**1.8-3.08**				**101.2-187.6**				**85.6-110.4**			
**Urinalysis**																								
**Individual number**	**Color**			**pH**			**Ketone**			**Bil**			**Oc.Bld**			**Urobil**								
	**pre**	**13**	**27**	**pre**	**13**	**27**	**pre**	**13**	**27**	**pre**	**13**	**27**	**pre**	**13**	**27**	**pre**	**13**	**27**						
**control**	**0.0**	**0.0**	**0.0**	**6.5**	**6.0**	**6.0**	**0.0**	**0.0**	**0.0**	**1.0**	**0.0**	**0.0**	**0.0**	**0.0**	**0.0**	**0.0**	**0.0**	**0.0**						
**1**	**0.0**	**0.0**	**0.0**	**8.5**	**7.5**	**7.5**	**0.0**	**0.0**	**0.0**	**1.0**	**1.0**	**0.0**	**0.0**	**0.0**	**4.0**	**0.0**	**0.0**	**0.0**						
**2**	**0.0**	**0.0**	**0.0**	**7.0**	**7.0**	**7.0**	**0.0**	**0.0**	**0.0**	**1.0**	**0.0**	**1.0**	**0.0**	**0.0**	**0.0**	**0.0**	**0.0**	**0.0**						
**3**	**0.0**	**0.0**	**0.0**	**8.0**	**8.0**	**6.5**	**0.0**	**0.0**	**0.0**	**1.0**	**0.0**	**1.0**	**0.0**	**0.0**	**0.0**	**0.0**	**0.0**	**1.0**						
**average**	**0.0**	**0.0**	**0.0**	**7.8**	**7.5**	**7.0**	**0.0**	**0.0**	**0.0**	**1.0**	**0.3**	**0.7**	**0.0**	**0.0**	**1.3**	**0.0**	**0.0**	**0.3**						
**S.D.**	**0.0**	**0.0**	**0.0**	**0.6**	**0.4**	**0.4**	**0.0**	**0.0**	**0.0**	**0.0**	**0.5**	**0.5**	**0.0**	**0.0**	**1.9**	**0.0**	**0.0**	**0.5**						
**Normal range**	**0.0**			**5.5-8.5**			**0-1**			**0-2**			**0-3**			**0-1**								
**Urinalysis**																								
**Individual number**	**U.Vol**			**S.Grav**			**Protein(mg/dL)**			**Glucose (mg/dL)**			**Na (mEq/L)**			**K (mEq/L)**			**Cl (mEq/L)**					
	**pre**	**13**	**27**	**pre**	**13**	**27**	**pre**	**13**	**27**	**pre**	**13**	**27**	**pre**	**13**	**27**	**pre**	**13**	**27**	**pre**	**7**	**14**			
**control**	**140**	**240**	**98**	**1.047**	**1.030**	**1.050**	**13.4**	**7.6**	**24.2**	**8.0**	**8.0**	**9.0**	**55**	**37**	**48**	**195.6**	**140.1**	**248.8**	**117**	**54**	**113**			
**1**	**60**	**60**	**115**	**1.072**	**1.046**	**1.050**	**48.7**	**41.3**	**72.4**	**10.0**	**7.0**	**11.0**	**49**	**177**	**72**	**366.1**	**243.1**	**270.9**	**260**	**116**	**151**			
**2**	**100**	**140**	**135**	**1.060**	**1.037**	**1.050**	**12.4**	**17.1**	**15.2**	**10.0**	**14.0**	**10.0**	**86**	**48**	**76**	**285.7**	**185.5**	**259.6**	**171**	**80**	**126**			
**3**	**170**	**180**	**127**	**1.048**	**1.047**	**1.056**	**11.0**	**13.0**	**34.9**	**6.0**	**6.0**	**12.0**	**39**	**42**	**54**	**216.9**	**224.7**	**280.8**	**123**	**108**	**134**			
**average**	**110.0**	**126.7**	**125.7**	**1.0600**	**1.0433**	**1.0520**	**24.0**	**23.8**	**40.8**	**8.7**	**9.0**	**11.0**	**58.0**	**89.0**	**67.3**	**289.6**	**217.8**	**270.4**	**184.7**	**101.3**	**137.0**			
**S.D.**	**45.5**	**49.9**	**8.2**	**0.0098**	**0.0045**	**0.0028**	**17.5**	**12.5**	**23.7**	**1.9**	**3.6**	**0.8**	**20.2**	**62.3**	**9.6**	**61.0**	**24.0**	**8.7**	**56.8**	**15.4**	**10.4**			
**Normal range**	**4.4-264**			**1.0191-1.0891**			**0-90.51**			**1.8-16.2**			**0-165.8**			**79.1-502.7**			**24.6-336.2**					

*RBC* Erythorocyte count, *WBC* Lymphocyte count, *AST* Aspartate transminase, *ALT* Alanine transminase, *Ketone* Ketone body, *Bil* Biliyubin, *Oc. Bld* Occult blood, *Urobil* Urobilinogen, *U.Vol* Urine body, *S.Grav* Specific gravity

## Discussion

The isolated DPSCs from adult teeth are usually limited in their number; thus, it is essential for pulp regenerative cell therapy to expand the isolated primary DPSCs without altering their stem cell properties. Therefore, providing a suitable microenvironment/niche similar to their physiological condition is important. We previously showed that MDPSCs have a high regenerative potential [9, 30]. However, it is a challenge for the utility of MDPSCs to be cost-effective and safe for approval as a medical device. It has been demonstrated that MSCs cultured under hypoxic condition could improve their regenerative potential in variety of tissues [31, 32]. Improved potential of the hypoxia-preconditioned MSCs has been reported for different clinical applications including spinal cord and lung injury, hindlimb ischemia, and immune-deficient models due to improving their secretion of reparative factors [33, 34] and initiating autophagy [35]. Thus, in this study, to further develop the isolation and culture method without using the MDPSC isolation device for DPSC subsets with high-regenerative potential, DPSCs were cultured under stable hypoxic (5%) and pH condition which were confirmed by non-contact oxygen analyzer and pH meter. The hpDPSCs were examined whether it could replace MDPSCs in pulp regenerative cell therapy. Transplantation of hpDPSCs with G-CSF exhibited a high pulp regenerative potential in vivo similar to MDPSCs with G-CSF without significant difference and no evidence of toxicity or adverse events. We previously demonstrated the combinatorial effect of MDPSCs with G-CSF for pulp regeneration on localization and engraftment of transplanted cells in the root canal, migration, and anti-apoptosis [10]. We recently demonstrated a significantly higher expression of G-CSF receptor, G-CSFR in human hpDPSCs compared with MDPSCs (68.2%, 38.3%, respectively) (unpublished data). In the present in vitro study, hpDPSCs showed higher proliferation rate, shorter culture period, and higher migration activity compared with MDPSCs in the presence of G-CSF. The hpDPSCs were survived similarly to MDPSCs in the presence of G-CSF. These findings suggest that the higher G-CSFR expression of the hpDPSCs might be one of the promising factors in cell properties for pulp regenerative cell therapy using with G-CSF. Thus, hpDPSCs have the advantage in clinical application for pulp regenerative therapy.

Oxygen is an important factor in the microenvironment of the cells for proliferation and differentiation [36]. A level of 3 to 6% of O_2_ was found in the physiological condition of adult organs and tissues including dental pulp tissue [21]. HIF-1α is a master transcription factor in the low oxygen partial pressure and represents a hypoxia key downstream effector which is involved in proliferation, angiogenesis, metabolism, and apoptosis [37, 38]. Moreover, HIF-1α is playing an important role in the metabolism and behavior of MSCs to maintain the biological functions and survival of transplanted stem cells [38]. It has been shown that hypoxia (5% O_2_) improved the characteristics of human MSCs such as increased proliferation rate, inhibition of senescence, and enhanced regenerative potential [34, 39]. Recently, DPSCs under hypoxic condition exhibited a higher proliferation rate and increased stem cell properties [24, 40]. Incubation of DPCs at 1% O_2_ for 24 h could enhance proliferation rate and increased expression level of *HIF-1* and *CXCR4* in human dental pulp cells [41]. We previously demonstrated that MDPSCs were enriched for stem cells, having higher angiogenic and neurotrophic potential, and pulp regenerative potential compared to non-isolated DPSCs [9, 25]. Also, non-isolated human DPSCs demonstrated that 5% O_2_ significantly increased the proliferation rate, migration ability, and expression of stem cell markers (*CXCR4* and *G-CSFR*) by flowcytometry compared with normoxia [24]. The present investigation demonstrated a higher gene expression of HIF-1α in hpDPSCs in 5% O_2_ condition compared with MDPSCs in normoxia. Therefore, hypoxic preconditioning or activating expression of HIF-1α is important to improve DPSC therapeutic potential. Proliferation rate and mRNA expression level of *CXCR4* were also significant higher in hpDPSCs compared to MDPSCs. These findings suggested that low O_2_ microenvironment may be essential to maintain the stem cell properties of DPSCs.

Angiogenesis, neurite extension, and migration of resident stem cells from the surrounding tissue of the teeth have been shown to play an important role in mediating the functional recovery of pulp tissue after pulpectomy [10, 42]. Several reports indicate that hypoxic condition of MSCs can enhance vascular tube formation [43] and neurogenesis [44]. The previous in vivo studies of hypoxia-preconditioned MSCs demonstrated enhanced angiogenic cytokine secretion in a murine hind-limb ischemia model [45]. Hypoxic cultures of DPSCs have previously demonstrated higher expression of angiogenic/neurotrophic factors, *VEGF*, *NGF*, and *BDNF* compared with normoxia and its CM stimulated neurite extension [24]. In the present study, hpDPSCs demonstrated a significantly higher gene expression of an angiogenic factor, *GM-CSF*, compared to MDPSCs, although there was no difference in *VEGF* expression and tube formation. Moreover, hpDPSCs enhanced neurite extension with increased expression of neurotrophic factors, *NGF* and *BDNF* which are some of major factors responsible for the innervation of pulp [46]. There was no difference in angiogenic/neurogenic potential between hpDPSCs and MDPSCs in the present dog pulpectomized teeth. Thus, these findings suggest that hpDPSCs may replace MDPSCs by the enhanced angiogenic/neurotrophic potential.

It has been shown that DPCs and PDLCs cultured under hypoxic condition increased the stimulatory effect of the CM on migration [41, 47, 48]. The hpDPSC CM exhibited similar migratory activity toward G-CSF to MDPSC CM. These findings suggest that a similar migratory activity of hpDPSCs in respect to releasing migration paracrine factors to MDPSCs may be one of important factors for pulp regeneration.

The hypoxic condition significantly reduces apoptosis of MSCs in vitro [49]. Enhanced survival and retention of hypoxic preconditioned MSCs are reported after injection in a spinal cord injury [50], muscle [51], and a cerebral infarction [35]. The role of HIF-1α in cell viability and anti-apoptosis of MSCs has been suggested [52]. Furthermore, the upregulation of secretory proteins involved in inhibition of apoptosis including thymosin-beta, elongation factor 2, and ganoderan under hypoxic condition are demonstrated in MSCs under hypoxic conditions [52]. In the current study, the hpDPSCs expressed *HIF-1α* significantly higher than MDPSCs and could reduce apoptosis by G-CSF similarly to the MDPSCs. The CM of the hpDPSCs also reduced apoptotic activity of PDLCs, a representative of the resident cells in the tissue surrounding the teeth, similarly to the CM of MDPSCs. These findings suggested that the transplanted hpDPSCs together with G-CSF may well-survive, retain, and enhance survival of migrating resident stem cells similarly as the MDPSCs.

The effect of hypoxia (1 and 5% O_2_) on the immunomodulatory function of adipose tissue-derived MSCs has been studied, demonstrating an upregulation of the immunomodulatory molecules upon stimulation with pro-inflammatory cytokines [53]. Interferon (IFN)-γ is a potent pro-inflammatory cytokine that produced by multiple immune cells to plays an important role in both innate and adaptive immunity. We evaluated the immunomodulation effect of hpDPSCs under stimulation with IFN-γ compared to MDPSCs. IFN-γ enhanced the immunomodulatory functions of hpDPSCs and MDPSCs and induced the expression of *IDO*, *PTGE*, *TGF-β*, and *IL-6*, with no significant difference except for *PTGE*. These results indicated that both hpDPSCs and MDPSCs have similar immunomodulatory effect under pro-inflammatory cytokine stimuli to improve their functionality and therapeutic capacity for pulp regeneration.

## Conclusions

In conclusion, the present investigation demonstrated that the hpDPSCs exhibited the higher proliferation and migration abilities, although anti-apoptotic and immunomodulatory properties were identical. The efficacy of hpDPSCs for pulp regeneration was identical to that of MDPSCs. Thus, these results suggested the potential clinical utility of hpDPSCs for pulp regeneration.

## Supplementary Information

**Additional file 1: Supplemental Fig. 1.**

## Data Availability

All data generated or analyzed during this study are included in this published article and its supplemental information files.

## Abbreviations

BDNF: Brain-derived neurotrophic factor
CM: Conditioned medium
DPSCs: Dental pulp stem cell
G-CSF: Granulocyte colony-stimulating factor
GDNF: Glial cell-derived neurotrophic factor
GM-CSF: Granulocyte monocyte colony-stimulating factor
GMP: Good manufacturing practice
IDO: Indoleamine 2,3-dioxygenase
IFN-γ: Interferon gamma
MDPSC: Mobilized dental pulp stem cell
MSCs: Mesenchymal stem cells
NGF: Nerve growth factor
NO: Nitric oxide
PDLCs: Periodontal ligament cells
PTGE: Prostaglandin E synthase
RT-PCR: Real-time quantitative polymerase chain reaction
TGFβ1: Transforming growth factor-beta1
TRH-DE: Thyrotropin releasing hormone degrading enzyme
VEGF: Vascular endothelial growth factor
hpDPSCs: Hypoxia-preconditioned dental pulp stem cells
