# Supplementary material for: Characterization of stable hypoxia-preconditioned dental pulp stem cells compared with mobilized dental pulp stem cells for application for pulp regenerative therapy
Source: Stem Cell Res Ther. 2021 May 29;12:302. doi: 10.1186/s13287-021-02240-w (PMC8164249; doi:10.1186/s13287-021-02240-w)
Supplement: Supplementary file 1 — Additional file 1: Supplemental Fig. 1. [file 13287_2021_2240_MOESM1_ESM.pptx]

## Slide 1
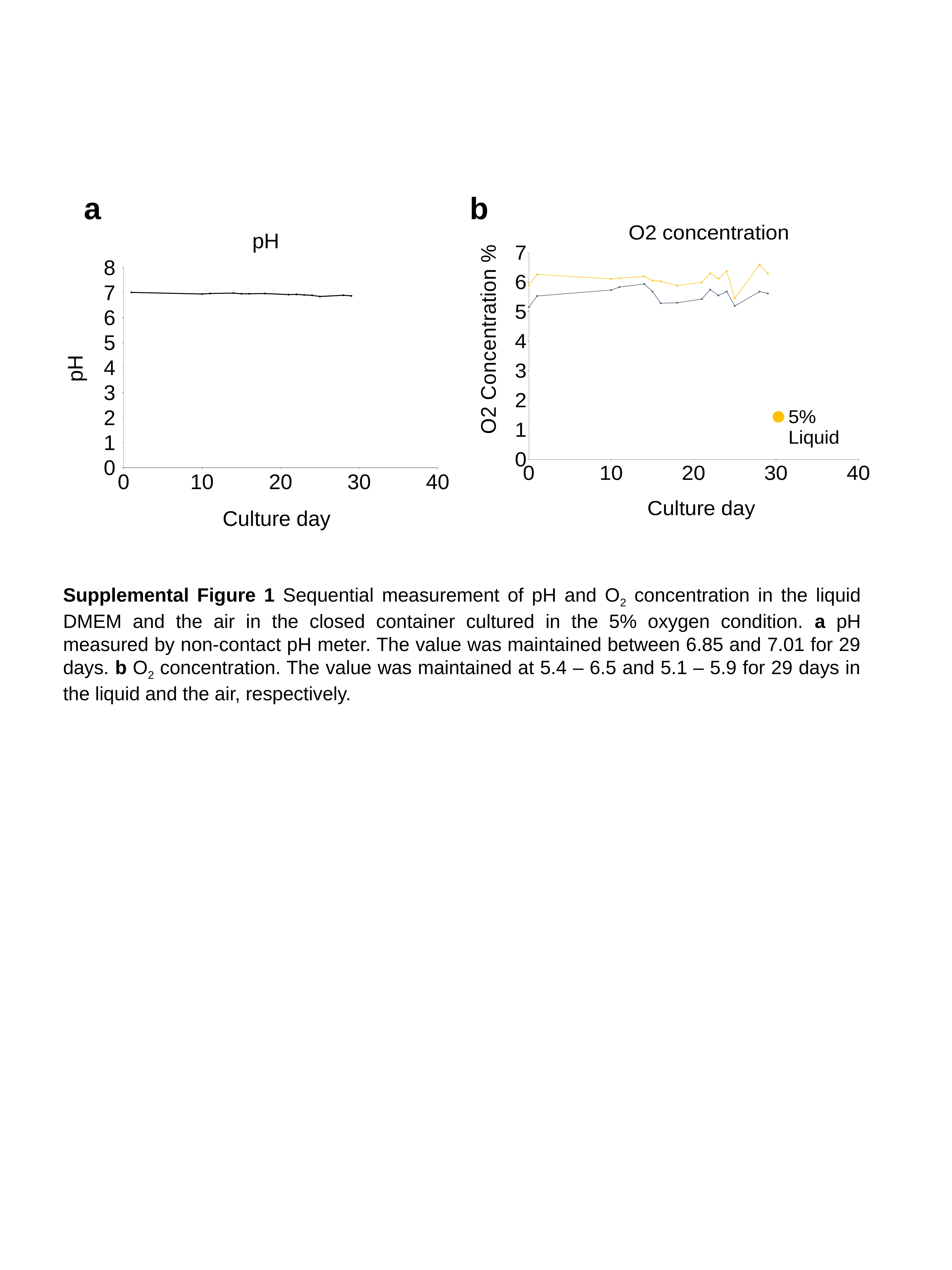

a
b
### Chart: O2 concentration
| Category | 5% Liquid | 5% Air |
|---|---|---|
### Chart:
| Category | pH |
|---|---|Supplemental Figure 1 Sequential measurement of pH and O2 concentration in the liquid DMEM and the air in the closed container cultured in the 5% oxygen condition. a pH measured by non-contact pH meter. The value was maintained between 6.85 and 7.01 for 29 days. b O2 concentration. The value was maintained at 5.4 – 6.5 and 5.1 – 5.9 for 29 days in the liquid and the air, respectively.
